# Local Defect Detection in Bearings in the Presence of Heavy-Tailed Noise and Spectral Overlapping of Informative and Non-Informative Impulses

**DOI:** 10.3390/s20226444

**Published:** 2020-11-11

**Authors:** Jakub Nowicki, Justyna Hebda-Sobkowicz, Radoslaw Zimroz, Agnieszka Wylomanska

**Affiliations:** 1 Faculty of Geoengineering, Mining and Geology, Wroclaw University of Science and Technology, Na Grobli 15, 50-421 Wroclaw, Poland; jakub.nowicki@pwr.edu.pl (J.N.); radoslaw.zimroz@pwr.edu.pl (R.Z.); 2 Faculty of Pure and Applied Mathematics, Wroclaw University of Science and Technology, Wybrzeże Wyspiańskiego 27, 50-370 Wroclaw, Poland; agnieszka.wylomanska@pwr.edu.pl

**Keywords:** local damage detection, vibration, heavy-tailed noise, informative and non-informative components spectral overlapping, dependency measures

## Abstract

The problem of the informative frequency band (IFB) selection for local fault detection is considered in the paper. There are various approaches that are very effective in this issue. Most of the techniques are vibration-based and they are related to the cyclic impulses detection (associated with the local fault) in the background noise. However, when the background noise in the vibration signal has non-Gaussian impulsive behavior, the classical methods seem to be insufficient. Recently, new techniques were proposed by several authors and interesting approaches were tested for different non-Gaussian signals. We demonstrate the comparative analysis related to the results for three selected techniques proposed in recent years, namely the Alpha selector, Conditional Variance-based selector, and Spearman selector. The techniques seem to be effective for the IFB selection for the non-Gaussian distributed vibration signals. The main purpose of this article is to investigate how spectral overlapping of informative and non-informative impulsive components will affect diagnostic procedures. According to our knowledge, this problem was not considered in the literature for the non-Gaussian signals. Nevertheless, as we demonstrated by the simulations, the level of overlapping and the location of a center frequency of the mentioned frequency bands have a significant influence on the behavior of the considered selectors. The discussion about the effectiveness of each analyzed method is conducted. The considered problem is supported by real-world examples.

## 1. Introduction

Local damage detection in bearings, using vibrations captured in industrial applications, requires an advanced pre-processing technique [1,2]. It may cover signal decomposition, modeling, analysis, filtering, enveloping, spectral analysis of envelope signal, and finally damage identification [3,4,5,6,7]. Most of the techniques usually assumed the different nature of the informative and non-informative components of the signal (impulsive/cyclic vs. deterministic or random, Gaussian vs. non-Gaussian, stationary vs. non-stationary, etc.). Recently, research focus has been put on the presence of impulsive disturbance in the raw observation that makes signal processing challenge. The assumed difference in terms of energy, frequency band, statistical properties, etc. is no longer valid. Using impulsiveness-based detectors is not reasonable when both components are impulsive. Periodicity measure, exploiting auto-correlation function, under really high impulsive disturbances becomes unstable and provides no meaningful results. Recent works published, e.g., by Kruczek et al. [8], suggested using alternative measures of dependence (instead of auto-correlation), see also [9,10]. In the literature, the special attention is paid to the fractional lower-order correlation analysis, where the fractional moments and fractional dependency measures are examined as the alternative statistics to the classical auto-correlation function [11,12,13,14,15]. The other interesting approach is based on the entropy in time and spectral domain [16,17,18]. This approach was successfully used in the impulsive signals analysis and also in the technical diagnostics [19,20,21,22], see also [23,24,25]. One can also find some dedicated diagnostic algorithms for impulsive signals analysis [26,27,28,29,30,31,32,33], but this problem is still open for deeper investigation [34,35] and more advanced techniques need to be proposed.

A novelty of this paper is related to a specific problem that may appear in a machine with a complex design (possible sources with different vibration signature) and non-stationary operating conditions with impulsive nature (as cutting, crushing, sieving, etc.) [36,37,38,39]. The impulsive disturbance is broadband, it excites the resonance frequency of machine and it is present in a wide frequency band. Let us assume there are present two different damages in the machine, both local and impulsive (so broadband) but with smaller energy (thus, not so broadband as disturbances). In most of the cases, the damage-related excitation and disturbance-related excitation will affect similar or even the same frequency band. The location of damage in the frequency domain may be different depending on machine geometry—or design factors in general. Therefore, the center frequency of disturbance will be the same (as the natural frequency of the structure), however, the center frequency of damage may depend on damage location (what bearing) and fault type (outer/inner race). The width of disturbance in the frequency domain is related to the nature of the process—in our case, it may change according to the impact caused by a falling piece of the ore and it has a random behavior. The width of the fault spectral signature may depend on load, fault size and type, the overall level of noise, etc.

In consequence, in the case of impulsive disturbance and impulsive content related to local damage, one may notice spectral overlapping of these components. Our experience shows that damage related excitation may be overlapped spectrally completely, partially, or in some cases may occupy different bands. How does spectral overlapping affect diagnostic procedures? In general, most of the research papers related to pre-processing of vibration signal before envelope analysis consider “selection” of informative frequency band and ignore other frequencies via filtering [4,5,40,41,42]. The level of spectral overlapping and locations of corresponding center frequencies will result in different level of noise in the extracted signal. It may happen that, after pre-filtering, the non-informative impulsive noise will remain in the signal. Application of well-known techniques based on kurtosis or other outliers-sensitive statistics will tune the informative frequency band (IFB) selector to non-informative components as they have much higher amplitudes (if not, there is no reason to search for new methods). The main purpose of this article is to investigate how spectral overlapping of informative and non-informative impulsive components will affect the diagnostic procedure. We are aware that for full overlapping we will not be able to remove impulsive noise completely, but we believe that a significant decrease in its amplitudes will be sufficient to use even classical techniques.

In the paper, we illustrate the problem using four cases, with three recently proposed techniques applied to such a problem for the first time. We consider the Alpha selector [43], Conditional Variance- based (CVB) selector [35], and Spearman selector [44] proposed as the most effective techniques for the IFB identification in the case of a strongly non-Gaussian distributed impulsive signal. We compare results in time and time-frequency domains by visual inspection and finally, we use—defined in [34]—an envelope spectrum-based feature to evaluate the quality of the applied techniques.

Finally, we present a kind of generalization (simulation-based) of the problem in two-dimensional maps that show how spectral overlapping may influence diagnostic results. The simulation results are supported by the discussion related to the real examples.

The rest of the paper is as follows: in Section 2, we describe the problem and discuss three considered techniques of IFB selection recently proposed for non-Gaussian vibration signals and the indicator for the evaluation of the selectors’ results. Next, in Section 3, we present the numerical results for four simulated signals representing different cases of the location of center frequencies related to cyclic and non-cyclic impulses in order to demonstrate how the spectral overlapping of the corresponding frequency bands influences the results of the diagnostic techniques. In Section 4, we present the results for a real signal while in Section 5 we discuss the real examples where the spectral overlapping occurs and present the Monte Carlo simulations. The last section concludes the paper.

## 2. Methodology

### 2.1. Problem Formulation

**Figure 1:**
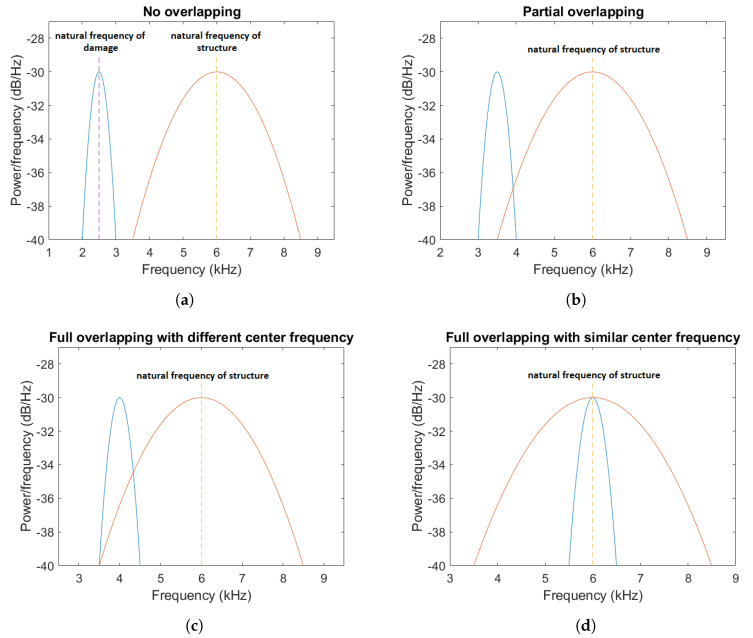
Different cases of the center frequency of a local fault placement with no overlapping frequency bands of cyclic and non-cyclic impulses (**a**), partial overlapping (**b**), and full overlapping (**c**,**d**).

A research goal of this work was to extract an informative signal from noisy vibration with heavy-tailed characteristics due to the production process. As mentioned, we assume fixed center frequency for non-cyclic broadband (impulsive) disturbances and several locations of center frequency for informative (cyclic) broadband (impulsive) components. As was mentioned, the location and the center frequency of damage in the frequency domain may change, depending on different factors. Thus, one may notice the spectral overlapping of cyclic and non-cyclic components. The illustrative plots demonstrating possible cases are presented in Figure 1a–d. The fault-related excitation may be overlapped spectrally completely (see Figure 1c,d), partially (see Figure 1b), or in some cases may occupy different bands (see Figure 1a).

We will use three selectors for IFB (Alpha selector, Conditional Variance-based selector, and Spearman selector) that were recently proposed as the effective indicators of the frequency band corresponding to informative signal for the strongly non-Gaussian time series. In our analysis, we check how the overlapping of the IFB and frequency of non-cyclic impulses influence the results but also investigate how the amplitude of non-cyclic impulses affect the ability of extraction as selectively as possible the signal of interest. First, we discuss the three techniques used in the further analysis.

### 2.2. Alpha Selector

The informative frequency band selectors are commonly used for the selection of the carrier frequency of the local fault basing on the representation of the signal in the time-frequency domain. Usually, the spectrogram is used here [45]. One of the known selectors, which has been successfully used in the case of the impulsive noise, is the Alpha selector [43,46]. This selector utilizes the advantage of the α-stable distribution, see more in [47]. The α-stable distribution has been used for vibration data pre-processing in [9,48].

The α-stable-distributed (denoted also as stable) random variable *X* is defined via the characteristic function $\phi_{X}\left(\theta \right)=E\left[\exp i\theta X\right]$ in the following way:

$$\begin{array}{c} \phi_{X}\left(u\right)=\left\{\begin{array}{cc} e^{-\sigma^{\alpha }{\left|u\right|}^{\alpha }\left\{1-i\beta \mathrm{sign}\left(u\right)\tan\left(\pi \alpha /2\right)\right\}+i\mu u}, & \alpha \neq 1\\ e^{-\sigma |u|\{1+i\beta \mathrm{sign}\left(u\right)\frac{2}{\pi }\log\left(|u|\right\}+i\mu u}, & \alpha =1, \end{array}\right. \end{array}$$

where stability index α∈(0,2], the scale parameter σ>0, the skewness β∈[−1,1], and shift μ∈R.

One can conclude the Gaussian distribution $N(\mu_{1},\sigma_{1})$ is a stable one with α=2, $\sigma =2\sigma_{1}^{2}$, and $\mu =\mu_{1}$. The β parameter is unimportant here. The important properties of the α-stable distributions can be found for instance in [47]. The α-parameter reflects the impulsiveness of the signal, the more impulsive signal is, the smaller the α parameter. The estimator of the α is not so sensitive to outliers as it holds, for example, in the case of sample kurtosis, the well-known measure of impulsiveness. What is more, the estimate of α will not change according to the increasing number of the impulses that appeared in the signal, while sample kurtosis is sensitive for changing impulses’ frequency and can give the misleading results if the number of the impulses increases.

The usage of the Alpha selector for bearing fault diagnosis can be found in [34,35]. The Alpha selector is constructed as follows: first, the signal is transformed into a time-frequency domain, then for the individual frequency band *f* we calculate the following statistic:

(1) $$A(f)=2-\hat{\alpha }\left(f\right),$$

where $\hat{\alpha }\left(f\right)$ is the estimator of α parameter for the time series corresponding to frequency band *f* in time-frequency representation. The values $A\left(f_{1}\right),A\left(F_{2}\right),\cdots ,A\left(f_{K}\right)$ (K-number of the frequency bins in time-frequency representation) along the frequency bands $f_{1},f_{2},\cdots ,f_{K}$ create the Alpha selector.

The value of α oscillates between 0 and 2—the smaller its value, the more impulsive the signal. As has been already mentioned, the parameter α=2 corresponds exactly to the Gaussian case, and the value of the Alpha selector tends to 0, otherwise, the selector becomes close to 1.

One can find various algorithms for α parameter estimation; see, e.g., [49,50]. Here we applied the method proposed by McCulloch, [51].

As has been presented in [34], the Alpha selector is efficient for the fault detection problem in the case of the heavy-tailed noise; however, in case the amplitude of the impulses grows, then the stability index α adapts to this and as suspected it decreases—thus, the selector increases and can react on the high-energy non-cyclic impulses much more than for the cyclic impulses related to the fault. It should be highlighted, the Alpha selector reacts only for the impulsive behavior of the signal and it does not detect its periodicity.

### 2.3. CVB Selector

For the idea of the selector based on the conditional variance statistic raised for technical (the local fault) diagnosis in the industrial crushing machine with heavy-tailed background noise, which is an inherent drawback of the machine’s operation, see [34]. The conditional variance statistic is related to the so-called 20/60/20 Rule [52,53]. According to this rule, if the sorted (in ascending order) Gaussian random sample is divided into three groups, the first one containing 20% of the largest values, the second one with 60% of the middle, and the last one with 20% of the smallest values, there is a particular relationship between some empirical characteristics corresponding to these groups, see [52]. What is interesting, a different number of partitions (more than 3) can be considered and the particular relationship between the groups is still preserved. In the literature [34], the CVB selector of the IFB has been defined as follows:

(2) $$\begin{array}{c} {\hat{C}}_{7}\left(x\right):={\left(\frac{{\hat{\sigma }}_{P_{3}}^{2}-{\hat{\sigma }}_{P_{4}}^{2}}{\hat{\sigma }}+\frac{{\hat{\sigma }}_{P_{5}}^{2}-{\hat{\sigma }}_{P_{4}}^{2}}{\hat{\sigma }}\right)}^{2}\sqrt{n}, \end{array}$$

where *n* is the sample size. The index 7 denotes the number of to the partitions applied to the vector $x=(x_{1},...,x_{n})$ while $P_{i}$ is the i-th set of this partition [53]:

(3) $$\begin{array}{c} {\hat{P}}_{1}:=\left(-\infty ,\;{\hat{x}}_{.004}\right],\;{\hat{P}}_{2}:=\left({\hat{x}}_{.004},\;{\hat{x}}_{.062}\right],\;{\hat{P}}_{3}:=\left({\hat{x}}_{.062},\;{\hat{x}}_{.308}\right],\;{\hat{P}}_{4}:=\left({\hat{x}}_{.308},\;{\hat{x}}_{.692}\right],\\ {\hat{P}}_{5}:=\left({\hat{x}}_{.692},\;{\hat{x}}_{.938}\right],\;{\hat{P}}_{6}:=\left({\hat{x}}_{.938},\;{\hat{x}}_{.996}\right],\;{\hat{P}}_{7}:=\left({\hat{x}}_{.996},\;\infty ,\right) \end{array}$$

where, ${\hat{x}}_{q}$ denotes the empirical quantile of order *q* for the vector $x=(x_{1},...,x_{n})$. The ${\hat{\sigma }}_{P_{i}}$ in (3) is the sample standard deviation in the set $P_{i}$ and $\hat{\sigma }$ is the sample standard deviation of the whole vector *x*. The main property of the division is that the variances corresponding to the appropriate sets are equal. If we assume the Gaussian distribution of the random vector, then the following is satisfied [53]:

(4) $$\sigma_{P_{1}}^{2}=\sigma_{P_{2}}^{2}=\sigma_{P_{3}}^{2}=\sigma_{P_{4}}^{2}=\sigma_{P_{5}}^{2}=\sigma_{P_{6}}^{2}=\sigma_{P_{7}}^{2}.$$

The condition (4) represents the equilibrium (with respect to the dispersion) for the conditional random vectors.

The conditional variance based (CVB) selector is constructed as follows: first, the signal is transformed into the time-frequency domain, then for the time series corresponding to the individual frequency band $f_{i}$, we calculate the ${\hat{C}}_{7}\left(f_{i}\right)$ statistic. The vector of statistics ${\hat{C}}_{7}\left(f_{1}\right),{\hat{C}}_{7}\left(f_{2}\right),\cdots ,{\hat{C}}_{7}\left(f_{K}\right)$ (*K*-number of the frequency bins in time-frequency representation) along the frequency bands $f_{1},f_{2},\cdots ,f_{K}$ creates the CVB selector.

The CVB selector is not sensitive to high-energy impulses [34]. However, similar to the Alpha selector, it does not indicate the cyclic behavior of the signal.

### 2.4. Spearman Selector

As was mentioned, the specificity of local fault is not only impulsiveness but also the periodicity of the occurring impulses. The advantage of detecting the cyclic behavior in the frequency bands by different correlation coefficients has been already used in [44]. It has been shown in [44] that the usage of the well known Spearman correlation coefficient allows us to distinguish cyclic (informative) and non-cyclic (non-informative) impulses. There are also considered other correlation measures in application to the IFB selection, i.e., Pearson and Kendall correlations. However, it has been presented that the selector based on Pearson correlation is sensitive for the high-energy impulses. Whereas, the selector based on the Kendall correlation has similar efficiency as the Spearman selector. Nevertheless, the complexity and time of computing the Kendall selector work to its disadvantage. Therefore, Spearman selector seems to be the most prominent among these three mentioned and has been selected for further testing in this paper. In the literature, there are also considered other dependency measures that are effective for the periodic behavior identification for the impulsive signal with non-Gaussian distribution [8,9].

The Spearman correlation-based selector is the one-dimensional representation of the Integrated Spearman Correlation Matrix, see more in [44]. The key role in the Spearman selector is the Spearman rank correlation coefficient, which for vector (X,Y) has the following form [54,55]:

(5) $$r_{XY}=\frac{\mathrm{cov}(Q,W)}{\sigma_{Q}\sigma_{W}},$$

where cov(·,·) is the covariance function, (Q,W) is a random vector of ranks corresponding to (X,Y), $\sigma_{Q}$ and $\sigma_{W}$ are the standard deviations of variables *Q* and *W*. The empirical version of the Spearman correlation coefficient for the random bi-dimensional sample $\left(x_{1},y_{1}\right),\cdots ,\left(x_{n},y_{n}\right)$, being the realization of the random vector (X,Y), is defined as follows:

(6) $$r_{xy}=\frac{\frac{1}{n-1}\sum_{i=1}^{n}\left(q_{i}-\bar{q}\right)\left(w_{i}-\bar{w}\right)}{{\left[\frac{1}{n-1}\sum_{i=1}^{n}{\left(q_{i}-\bar{q}\right)}^{2}\frac{1}{n-1}\sum_{i=1}^{n}{\left(w_{i}-\bar{w}\right)}^{2}\right]}^{1/2}},$$

where $q_{i},w_{i}$ are the empirical counterparts of random variables *Q* and *W*, whereas $\bar{q}$ and $\bar{w}$ are sample means of the relevant rank samples.

Spearman correlation takes values between [−1,1] and tests a monotonic relationship. In the case of the Spearman selector of the IFB, the relationship of sub-signals from the spectrogram is tested and the pairs $\left(x_{i},y_{j}\right)$ in Equation (5) are replaced by corresponding elements of the considered sub-signals for given frequency bands. The final formula for the Spearman selector requires several earlier steps (calculating the dependence map, thresholding, dependence map integration). The details are described in [44].

As mentioned, the described above selectors have been considered for the issue related to local fault identification. However, according to our knowledge, they have not been tested in the problem considered in this paper. Namely, when the frequency bands corresponding to signal of interest (SOI) and the non-informative impulsive signal overlap. In the next section, we perform the comparative study for this specific problem.

### 2.5. Results Evaluation

To make the comparative study for tested selectors, we propose to use the Envelope Spectrum-based Indicator (ENVSI). It enables the numerical comparison of the envelope spectrum of filtered (by using different methods) signals. The ENVSI was defined in [34] and it takes the following form:

(7) $$ENVSI=\frac{\sum_{i=1}^{M_{1}}{AIS_{i}}^{2}}{\sum_{k=1}^{M_{2}}SES_{k}},$$

where $M_{1}$ is the number of fault components used for analysis while $M_{2}$ is the number of frequency bins applied to obtain the total energy. The $AIS_{1},AIS_{2},\cdots ,AIS_{M_{1}}$ in Equation (7) denote the amplitudes of the information signal represented by the components with the fault frequency while the vector $SES_{1},SES_{2},\cdots ,SES_{M_{2}}$ represents the square envelope spectrum corresponding to the frequencies $f_{1},f_{2},\cdots ,f_{M_{2}}$. As noted in [34], the amount of the harmonic components $M_{1}$ set to 10 seems to be sufficient to recognize the periodicity in the signal. The value of the parameter $M_{2}$ is strictly associated with value $M_{1}$ that corresponds to the frequency number to which the considered frequencies are summed up. In order to use the ENVSI, there is a need to know the frequency of the local fault, which is essential to consider an appropriate AISs with correct locations (repetitive impulses of fault). The higher the value of the ENVSI, the easier the fault diagnosis.

## 3. Numerical Analysis

In this part, we apply the techniques presented in Section 2.2 for the IFB selection for the simulated signals. The considered simulated signals are composed of three elements (the complete description has been presented in [34]):

- the Gaussian white noise that represents the background noise;
- the non-periodic impulses associated with characteristic machine work;
- the periodic impulses related to a local defect.

In our analysis, we investigate four signals: $s_{1}$, $s_{2}$, $s_{3}$, and $s_{4}$. They differ in the setting of a center frequency of cyclic impulses: $s_{1}$—2500 Hz, $s_{2}$—3500 Hz, $s_{3}$—4000 Hz, $s_{4}$—6000 Hz. For all considered signals, sampling frequency is 25 kHz. The other parameters of the simulated signals are the same, i.e., the center frequency of the non-periodic impulses—6000 Hz, the amplitude of the non-periodic impulses—20, and amplitude of the periodic impulses—3. The choice of these four different values of center frequency allows us to consider the following cases corresponding to the non-cyclic and cyclic impulses frequency bands locations:

- no overlapping;
- partial overlapping;
- full overlapping with different center frequency;
- full overlapping with similar center frequency.

The four considered cases correspond to the examples presented in Figure 1a–d respectively.

**Figure 2:**
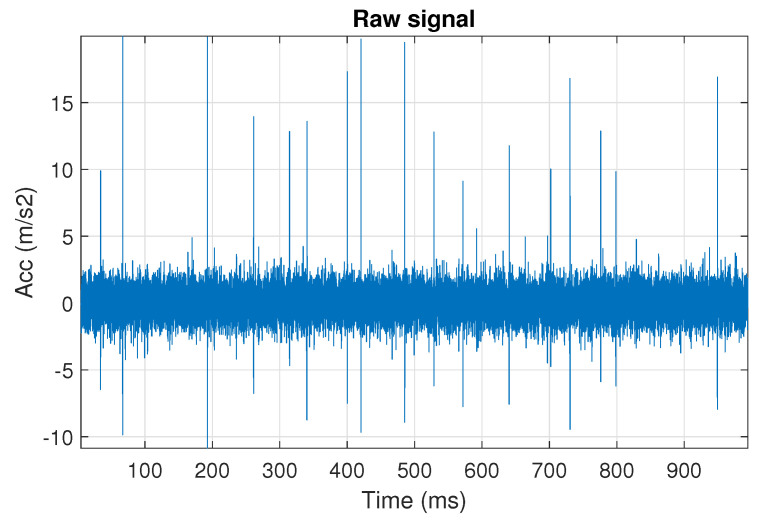
The simulated signal $s_{1}$, it corresponds to the case presented in Figure 1a.

**Figure 3:**
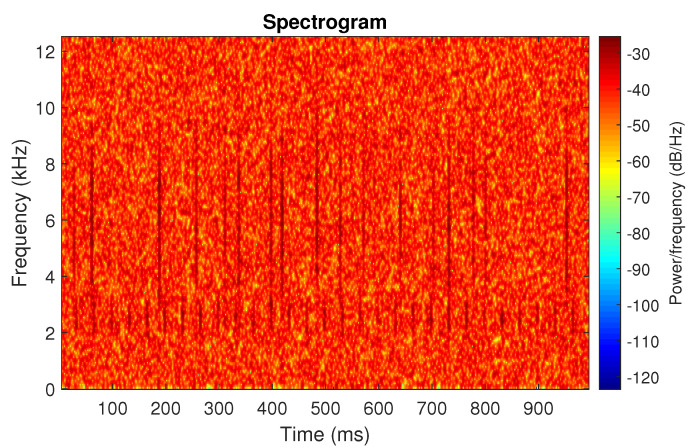
Spectrogram of the simulated signal $s_{1}$.

First, we examine the signal $s_{1}$, see Figure 2. The spectrogram of the signal is shown in Figure 3. All spectrograms have been obtained with a Hamming window [45] of length 256, with 217 $\left(\lfloor 85\%\cdot 256\rfloor \right)$ samples overlapping. The number of frequency points used to calculate the Fourier transform has been set to 512.

**Figure 4:**
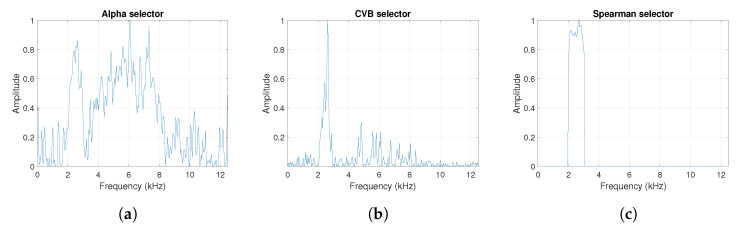
The considered informative frequency band (IFB) selectors for the simulated signal $s_{1}$. (**a**) Alpha selector. (**b**) CVB selector. (**c**) Spearman selector.

One can see the frequency band of non-periodic impulses, which is in the range of 3–8 kHz, is adjacent to the frequency band of periodic impulses, which occurs in the range of 2–3 kHz. This signal corresponds to the case presented in Figure 1a. The three selectors described in Equations (1), (2) and (6) have been applied. The corresponding final results have been shown in Figure 4. It is easy to notice, that not all considered methods work properly. It is obvious that all selectors correctly recognize the informative frequency band, but the Alpha selector (see Figure 4a) additionally points out the range of the band in which the non-periodic impulses occur. Both the CVB selector (Figure 4b) and the Spearman selector (Figure 4c) work properly, but the Spearman selector seems to be more selective. For the Spearman selector, we observe that its values outside the IFB range are equal to zero.

**Figure 5:**
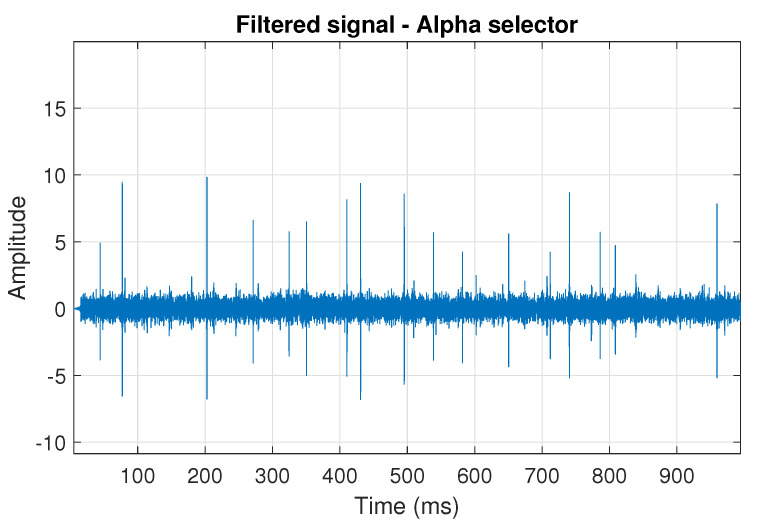
Filtered signal $s_{1}$ by Alpha selector.

**Figure 6:**
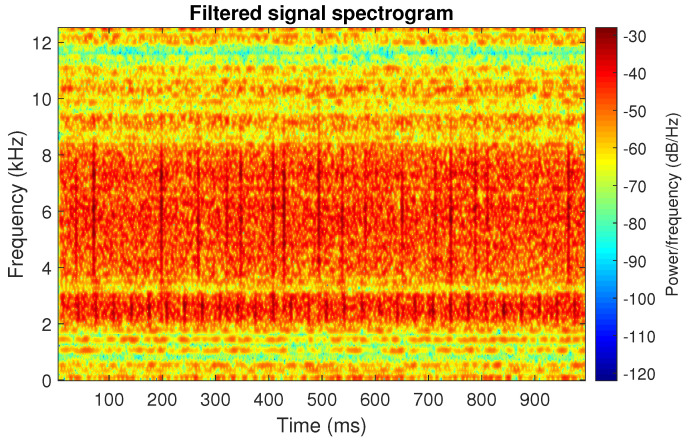
Spectrogram of filtered signal $s_{1}$ by Alpha selector.

The obtained selectors have been used to filter the signal $s_{1}$. The filtered signal by the Alpha selector has been shown in Figure 5 and its spectrogram is presented in Figure 6. This selector has high values not only for the carrier frequency of the cyclic impulses but also in the frequency band where non-cyclic impulses occur. This makes it different from the other considered selectors. It is less selective and the filtered signal contains much more noise.

**Figure 7:**
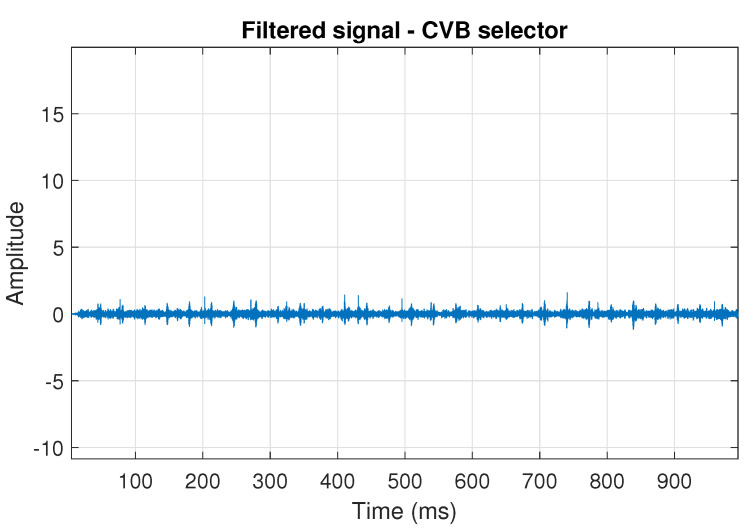
Filtered signal $s_{1}$ by CVB selector.

**Figure 8:**
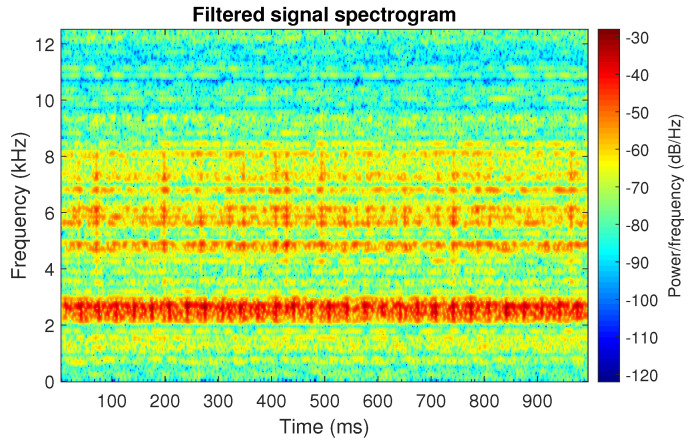
Spectrogram of filtered signal $s_{1}$ by Conditional Variance-based (CVB) selector.

**Figure 9:**
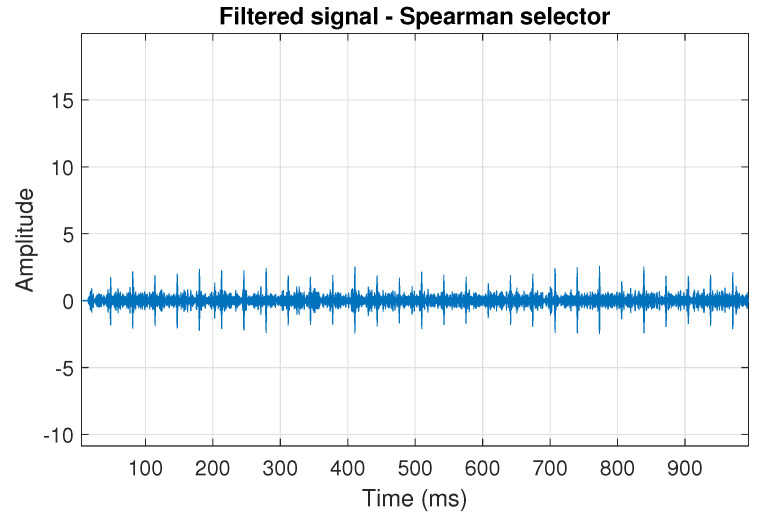
Filtered signal $s_{1}$ by Spearman selector.

**Figure 10:**
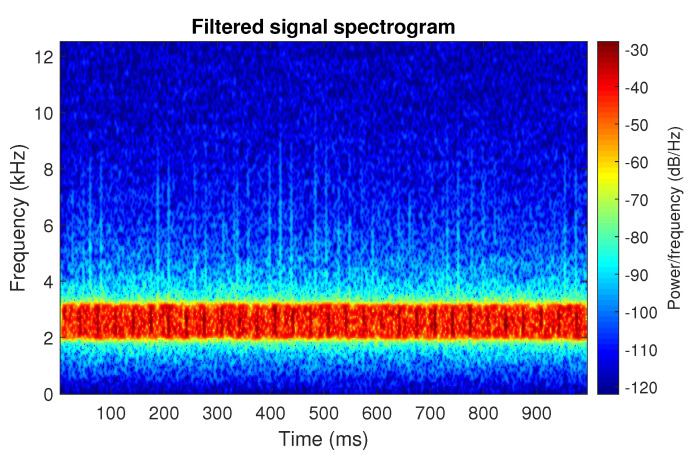
Spectrogram of filtered signal $s_{1}$ by Spearman selector.

The filtration results with the CVB selector have been presented in Figure 7. As one can see, the periodic impulses with amplitudes of about 1 are noticeable. The filtered signal does not contain high amplitude impulses associated with heavy-tailed noise. The spectrogram of the filtered signal indicates low energy for the frequency band in the range 3–8 kHz (see Figure 8). The signal filtered by the Spearman selector has been shown in Figure 9. Cyclic impulses with the amplitude of 2 are noticeable. Comparing it to the raw signal $s_{1}$, see Figure 2, one can notice, the components of raw signal related to Gaussian white noise and heavy-tailed noise have been almost completely removed. This is even more visible in the spectrogram of the filtered signal, see Figure 10.

**Figure 11:**
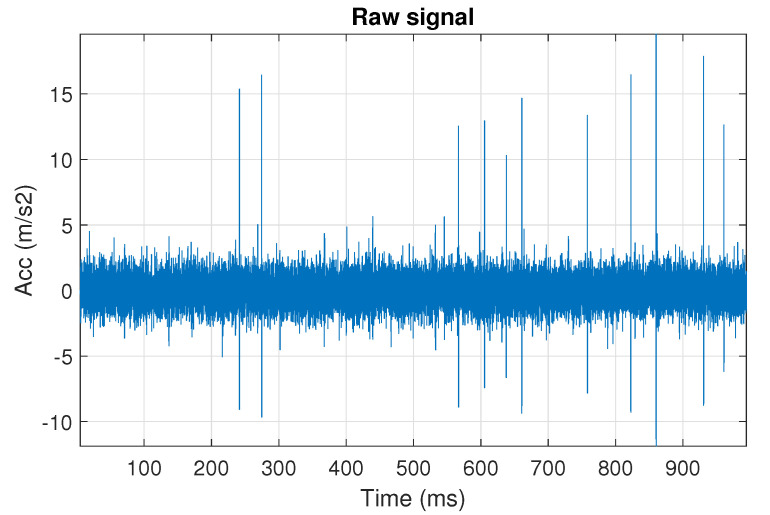
The simulated signal $s_{2}$. It corresponds to the case presented in Figure 1b.

**Figure 12:**
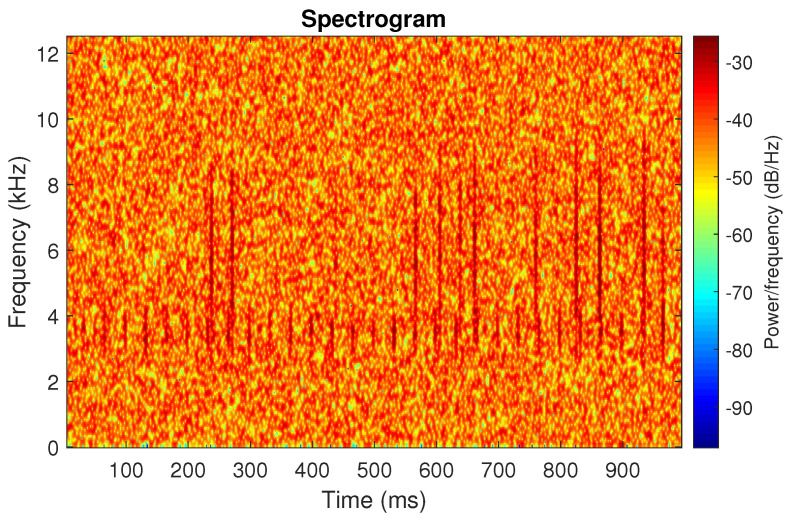
Spectrogram of the simulated signal $s_{2}$.

**Figure 13:**
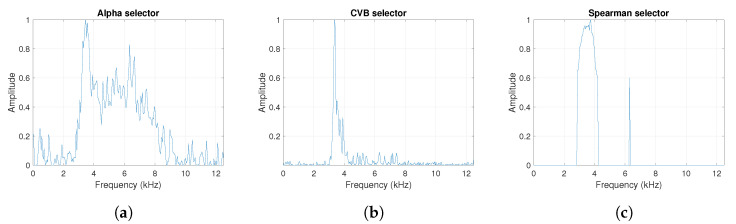
IFB selectors for the simulated signal $s_{2}$. (**a**) Alpha selector. (**b**) CVB selector. (**c**) Spearman selector.

**Figure 14:**
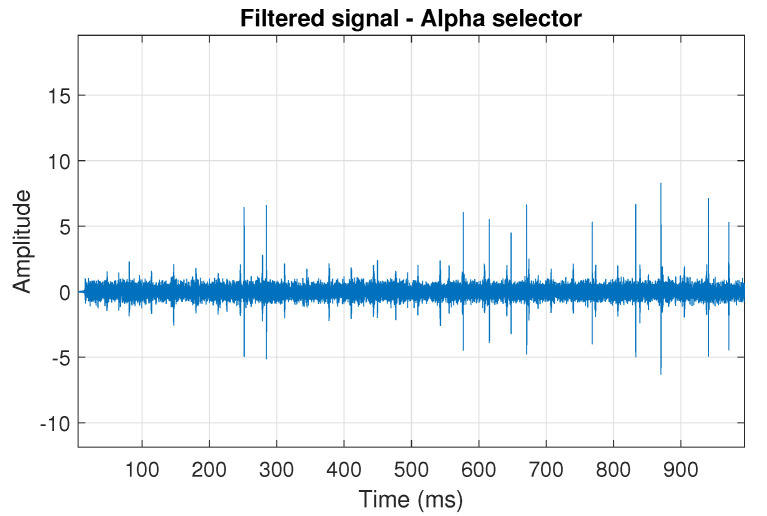
Filtered signal $s_{2}$ by Alpha selector.

**Figure 15:**
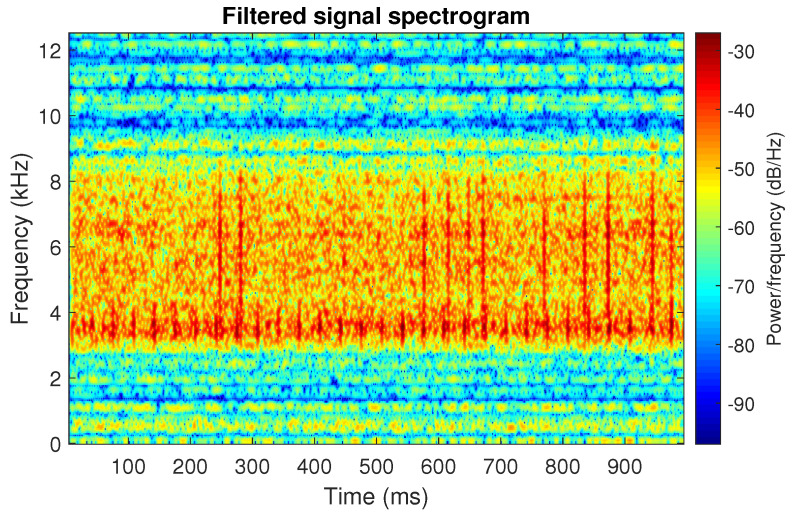
Spectrogram of filtered signal $s_{2}$ by Alpha selector.

**Figure 16:**
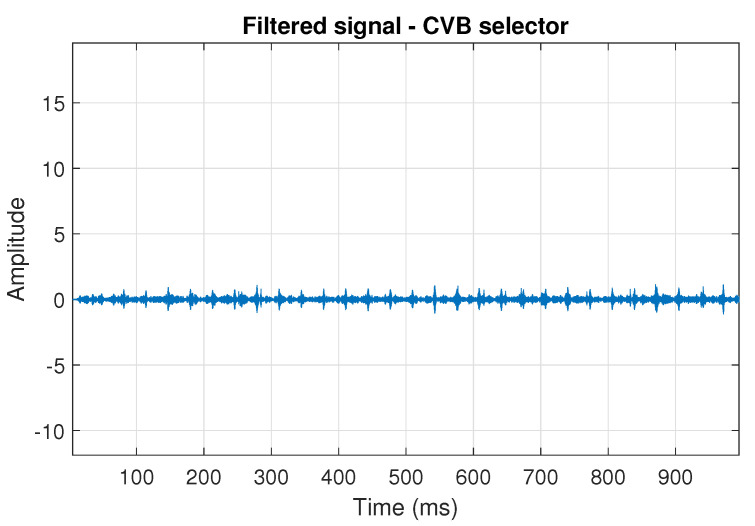
Filtered signal $s_{2}$ by CVB selector.

**Figure 17:**
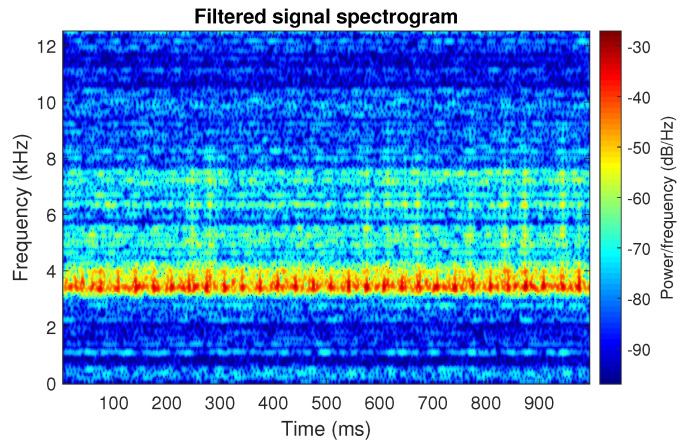
Spectrogram of filtered signal $s_{2}$ by CVB selector.

**Figure 18:**
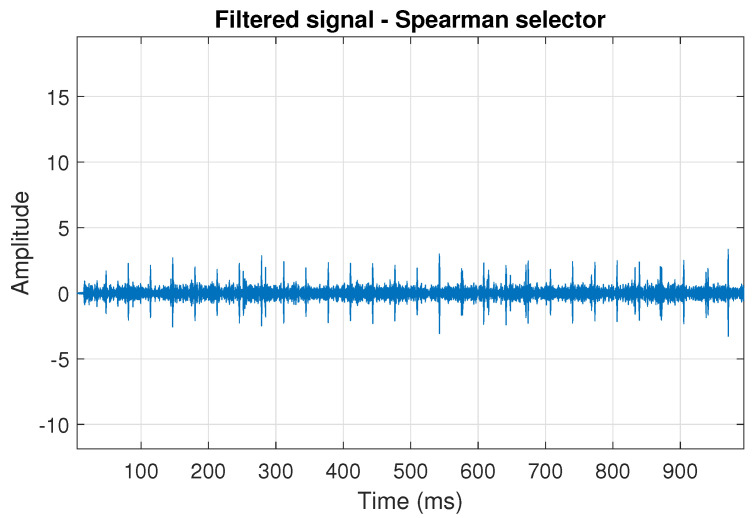
Filtered signal $s_{2}$ by Spearman selector.

**Figure 19:**
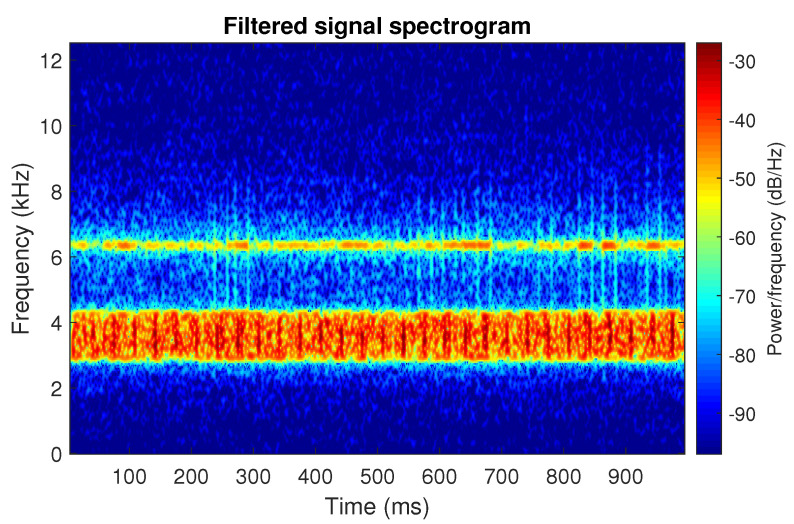
Spectrogram of filtered signal $s_{2}$ by Spearman selector.

Similar as for the signal $s_{1}$, we have performed the analysis for the signal $s_{2}$. The time series is shown in Figure 11 and its time-frequency representation is presented in Figure 12. In this case, the center frequency has been set to 3.5 kHz, so that the frequency band associated with the periodic impulses overlaps the band corresponding to the non-cyclic disturbances, but not completely. This situation corresponds to the case presented in Figure 1b. As one can see on the spectrogram (see Figure 12), these bands overlap in the range 3.5–4 kHz. Next, all three methods for IFB selection have been applied. The final results are filter characteristics based on the selectors, see Figure 13. As in the analysis of the signal $s_{1}$, also in this case each selector correctly points out the IFB. In comparison, the highest difference we observe for the Alpha selector (Figure 13a). For the signal $s_{1}$ this selector has high value also for the non-informative frequency band, while for the signal $s_{2}$ it does not have such high values as previously. Despite this, these values are still large compared to the CVB (Figure 13b) and Spearman (Figure 13c) selectors. The last two mentioned selectors are insensitive to non-periodic impulses and work properly, but the Spearman selector outperforms the CVB one. The value of the Spearman selector for the non-informative frequency band is equal to 0, due to used thresholding procedure proposed in [44]. One can see that the Spearman selector has a narrow peak around 6 kHz. This impulsive component does not has much influence on filtering because it is very narrow. Moreover, after minor tuning of thresholding it could be eliminated completely. Figure 14 and Figure 15 show the effect of filtering with the Alpha selector. The impulses associated with the heavy-tailed noise are still present, but they have a lower amplitude than in the raw signal. Periodic impulses related to the local defect gently emerge from the noise, and their amplitude is approximately 2. The signal $s_{2}$ after filtration with the CVB selector and its spectrogram, are very similar to the first considered case (see Figure 16 and Figure 17). Cyclic impulses with the amplitude of about 1 are visible, but there are no non-periodic impulses associated with the specificity of the machine’s operation. The filtered signal by the Spearman selector does not contain the non-periodic impulses with high amplitude, see Figure 18. As one can see on the spectrogram (see Figure 19), the frequency band in the range of 3–4 kHz has high energy. The energy is also high around a narrow band 6 kHz. It is a consequence of the fact that Spearman selector has a narrow but high amplitude at this place.

**Figure 20:**
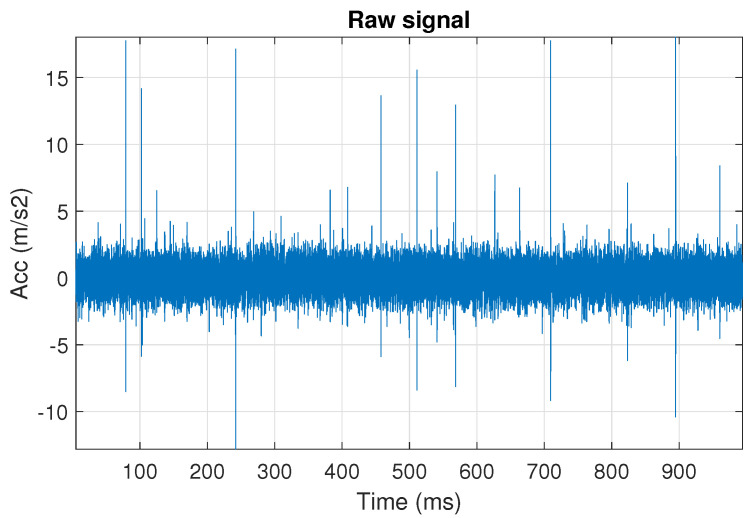
The simulated signal $s_{3}$. It corresponds to the case presented in Figure 1c.

**Figure 21:**
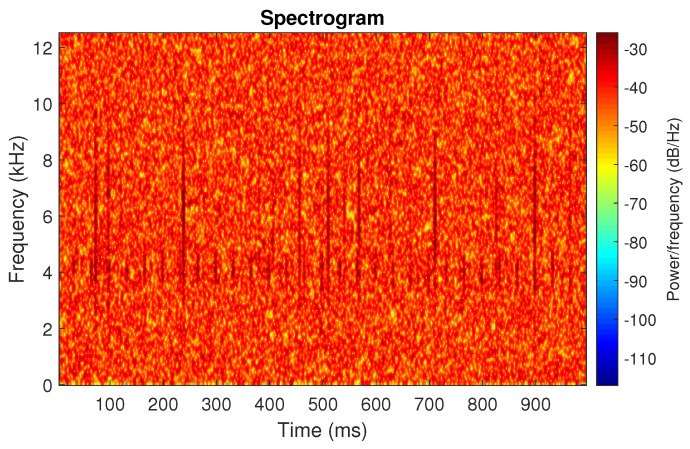
Spectrogram of the simulated signal $s_{3}$.

**Figure 22:**
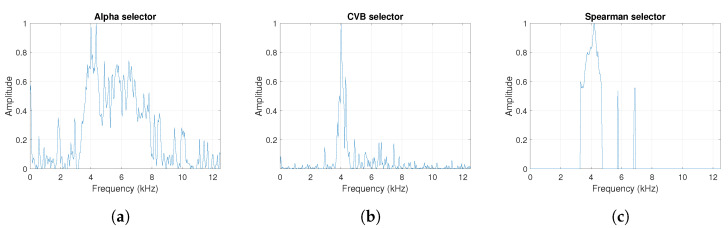
IFB selectors for the simulated signal $s_{3}$. (**a**)Alpha selector. (**b**) CVB selector. (**c**) Spearman selector.

**Figure 23:**
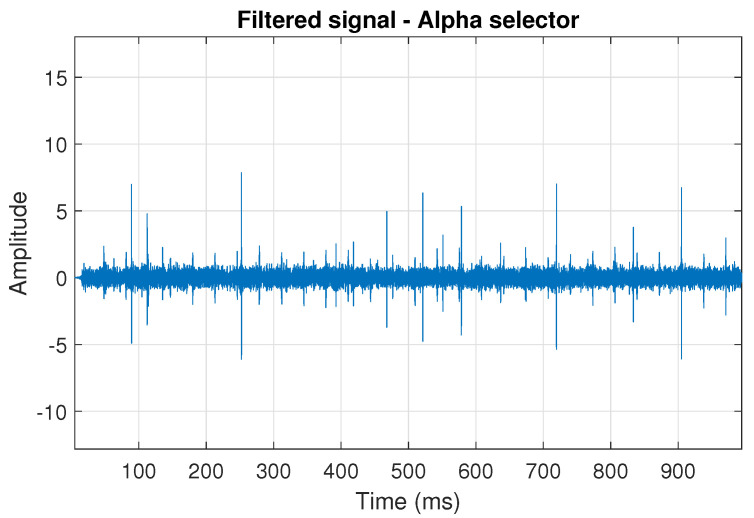
Filtered signal $s_{3}$ by Alpha selector.

**Figure 24:**
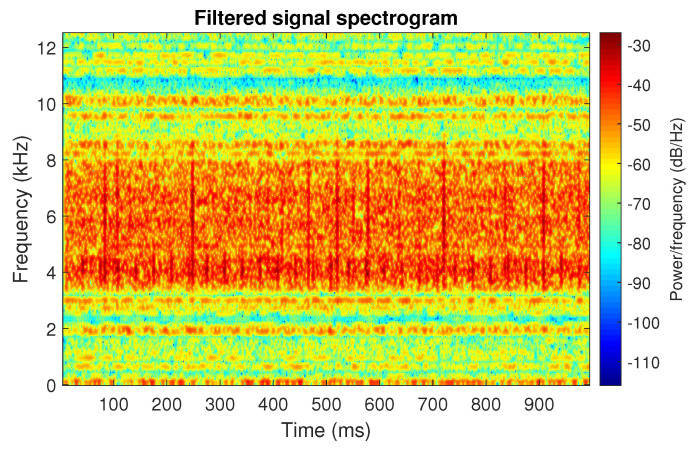
Spectrogram of filtered signal $s_{3}$ by Alpha selector.

**Figure 25:**
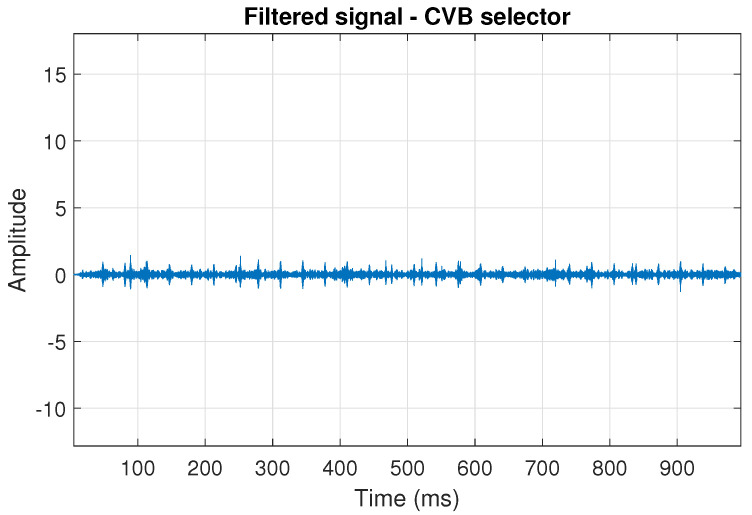
Filtered signal $s_{3}$ by CVB selector.

**Figure 26:**
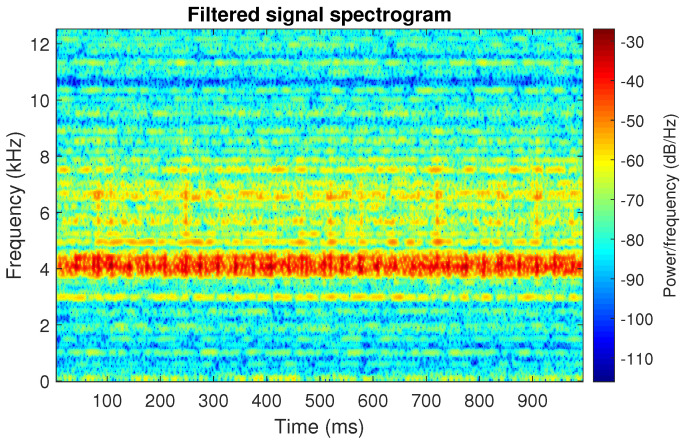
Spectrogram of filtered signal $s_{3}$ by CVB selector.

**Figure 27:**
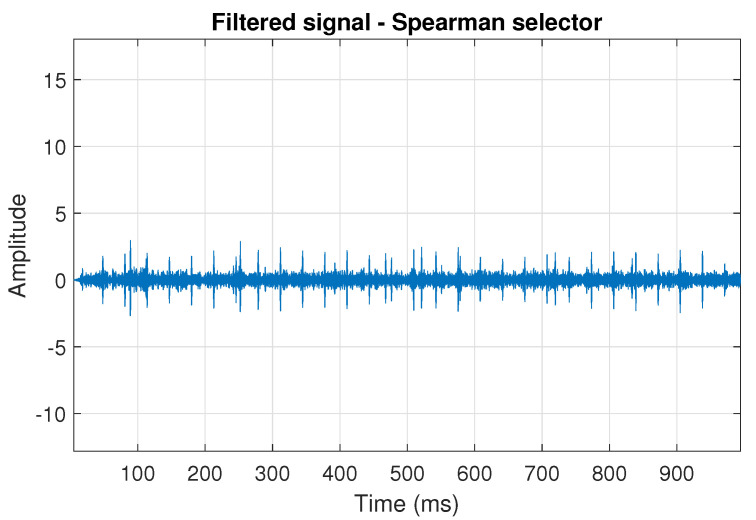
Filtered signal $s_{3}$ by Spearman selector.

**Figure 28:**
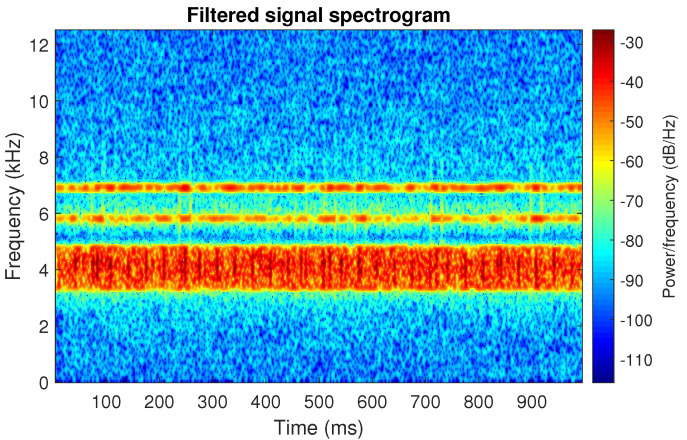
Spectrogram of filtered signal $s_{3}$ by Spearman selector.

In the third case, signal $s_{3}$ (see Figure 20) has been investigated, for which frequency bands of informative and non-informative impulses completely overlap, but their center frequencies are different (this situation has been shown in Figure 1c). Such a location of these bands is well visible on the spectrogram, see Figure 21. As one can see, periodic impulses are in the range of 3.5–4.5 kHz, while non-cyclic impulses occur in the range of 3.5–9 kHz. Using the time-frequency representation, the selectors have been calculated (see Figure 22). As in the previous cases, only the Alpha selector (Figure 22a) indicates high values in the non-informative frequency band (corresponding to the non-cyclic disturbances). All selectors correctly determine the band of occurrence of periodic impulses, see Figure 22a–c. It is worth noting, this band also includes heavy-tailed noise. The filtered signal by the Alpha selector and its spectrogram have been shown in Figure 23 and Figure 24, respectively. As one can see from the spectrogram, only the Gaussian white noise has been removed. Consequently, the filtered signal has both periodic and non-periodic impulses. The amplitude of the first ones is approximately 2, while the amplitude of the latter is approximately 5. Next, the signal $s_{3}$ has been filtered with the CVB selector. The periodic impulses are noticeable, but their amplitude is very low, it is about 1 (see Figure 25). The spectrogram of this signal has been shown in Figure 26. The filtered signal by Spearman selector has been presented in Figure 27. Periodic impulses have the amplitude of approximately 2.5. The spectrogram of the filtered signal has been shown in Figure 28.

**Figure 29:**
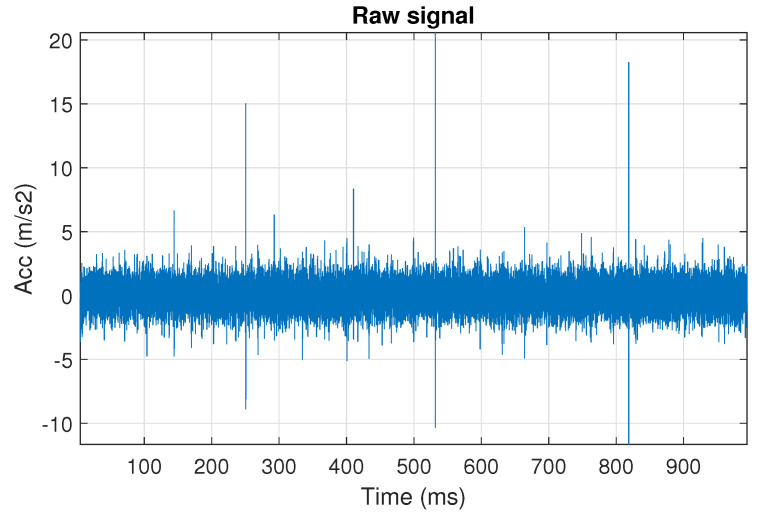
The simulated signal $s_{4}$. It corresponds to the case presented in Figure 1d.

**Figure 30:**
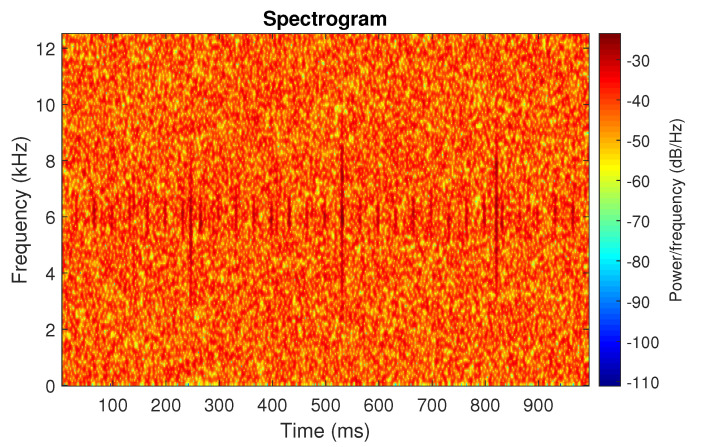
Spectrogram of the simulated signal $s_{4}$.

**Figure 31:**
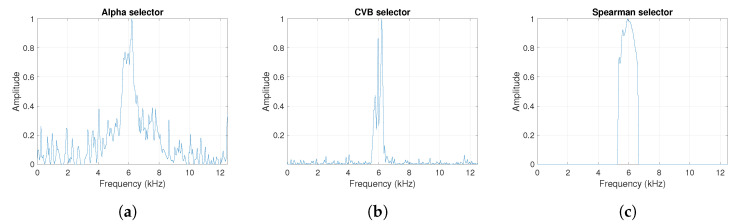
IFB selectors for the simulated signal $s_{4}$. (**a**) Alpha selector. (**b**) CVB selector. (**c**) Spearman selector.

**Figure 32:**
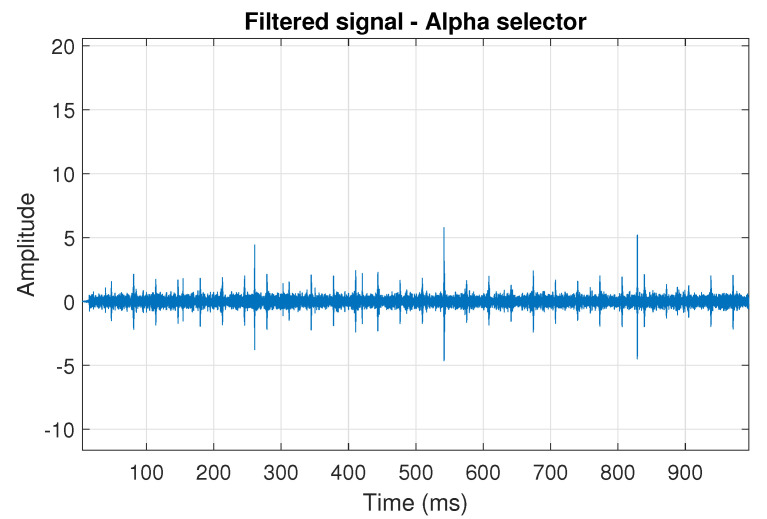
Filtered signal $s_{4}$ by Alpha selector.

**Figure 33:**
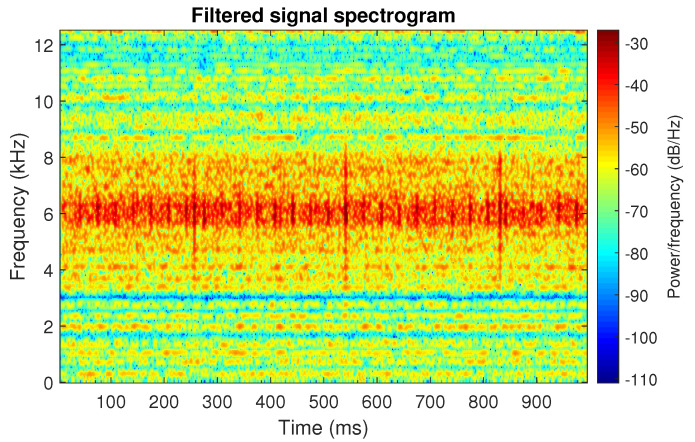
Spectrogram of filtered signal $s_{4}$ by Alpha selector.

**Figure 34:**
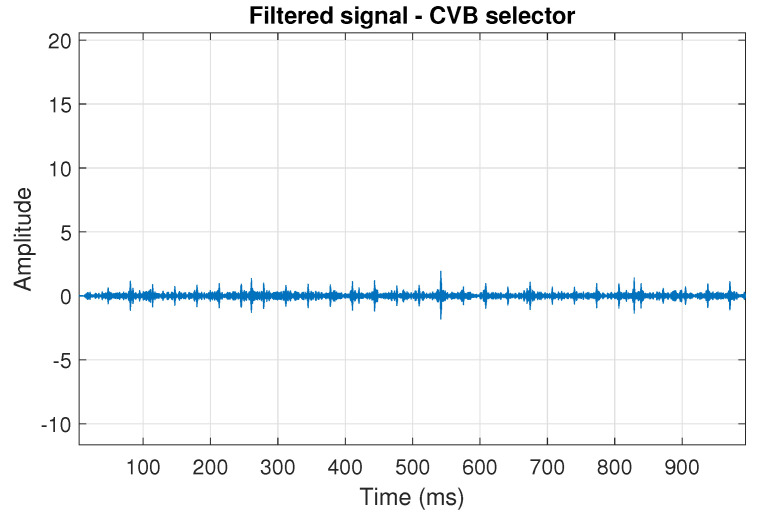
Filtered signal $s_{4}$ by CVB selector.

**Figure 35:**
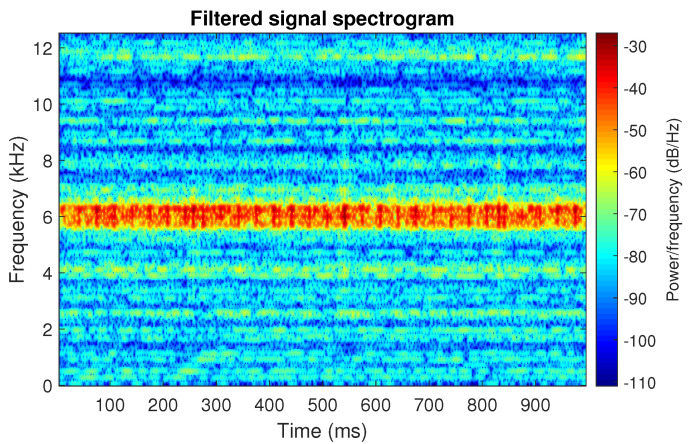
Spectrogram of filtered signal $s_{4}$ by CVB selector.

**Figure 36:**
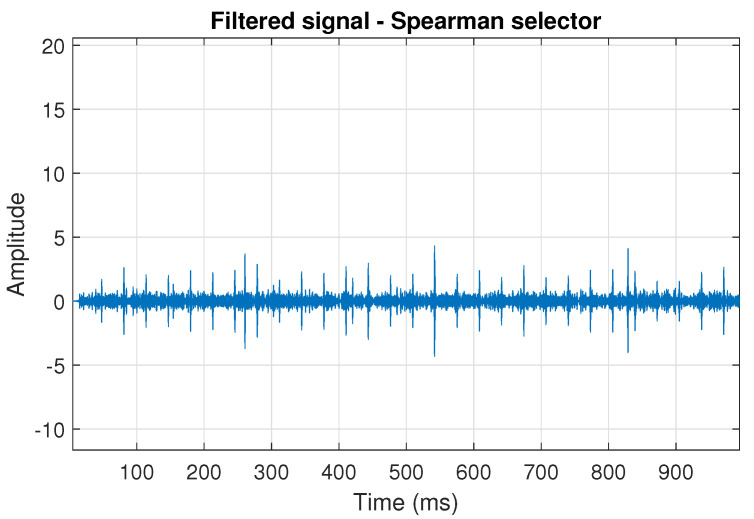
Filtered signal $s_{4}$ by Spearman selector.

**Figure 37:**
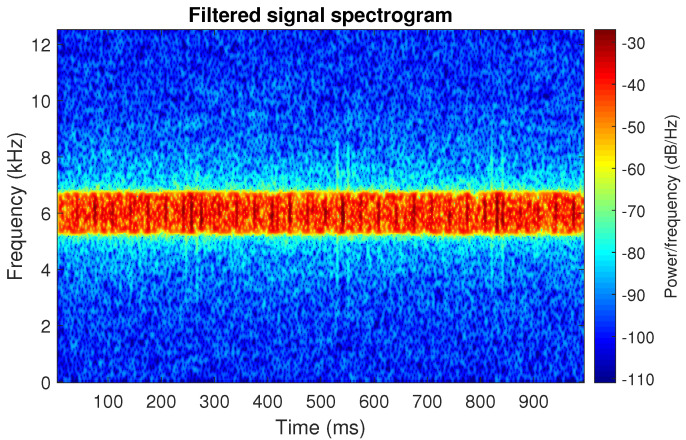
Spectrogram of filtered signal $s_{4}$ by Spearman selector.

The last simulated signal $s_{4}$ has been illustrated in Figure 29. It corresponds to the case presented in Figure 1d. Recall that the center frequency of IFB has been set to 6 kHz. As a result, the bands of periodic and non-periodic impulses completely overlap. This is visible in the spectrogram of this signal, see Figure 30. Then, the selectors have been determined using the three previously mentioned methods (see Figure 31). As one can see in Figure 32, Figure 33, Figure 34, Figure 35, Figure 36 and Figure 37, all selectors indicate the frequency band where the impulses associated with the fault occur. However, this band is not fully informative due to the fact that there are also non-periodic impulses. As one can see, for each of the filtered signals we can notice the periodicity. In the case of the signal filtered by the Alpha (illustrated in Figure 32) and Spearman selectors (presented in Figure 36), the amplitude of periodic impulses is approximately 2. After filtration with the CVB selector, their amplitude is about 1, see Figure 34. Comparing the spectrograms of these three filtered signals (see Figure 33, Figure 35 and Figure 37), one can conclude that the filtration with the Spearman selector cleans Gaussian white noise the most.

To compare the considered algorithms for IFB selection applied for each of the four cases, Table 1 has been created. It contains the values of the ENVSI indicator (see Section 2.5 for more details). As one can see, the approach based on the Spearman correlation has the highest index. It means that the Spearman selector indicates the informative frequency band in the most effective way in comparison to other selectors. For the CVB method, the values are very similar but slightly lower. The worst results we obtain are for the Alpha selector. The ENVS indicator values for this method differ significantly from the other results. It is worth noting that the values in the column corresponding to the Spearman selector decrease as the center frequency of the periodic impulses increases, i.e., with progressing overlap of the bands.

## 4. Investigation on Real Data

In practice, the investigation related to the influence of the spectral overlapping of the cyclic and non-cyclic impulses may be limited. Data from the same machine with faults in different frequency bands are hardly accessible, even inaccessible. However, in this section, the effectiveness of the considered methods is checked for the real signal, which is the case where the full overlapping of cyclic and non-cyclic impulses with similar center frequency occurs.

**Figure 38:**
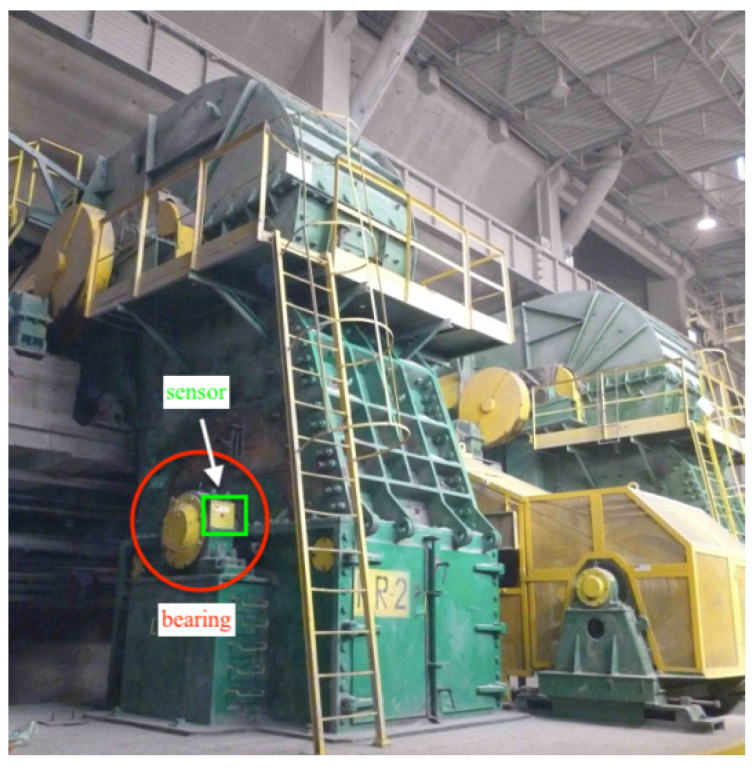
Hammer crusher with bearing and sensor locations marked.

This signal has been obtained from a copper ore crusher, see Figure 38, that had a damaged bearing. The vibration signals have been collected using Endevco accelerometers, whereas a shaft speed was measured with a BruelKjaer laser probe. The collected data have been recorded on the NI DAQ card using Labview Signal Express Software. The signal consists of 250,000 samples that were obtained in 10 s (sampling frequency is 25,000 Hz).

**Figure 39:**
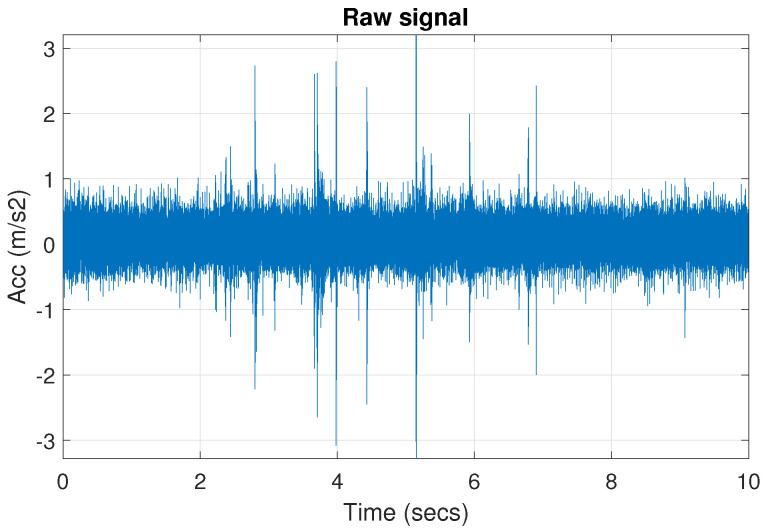
The real signal r.

**Figure 40:**
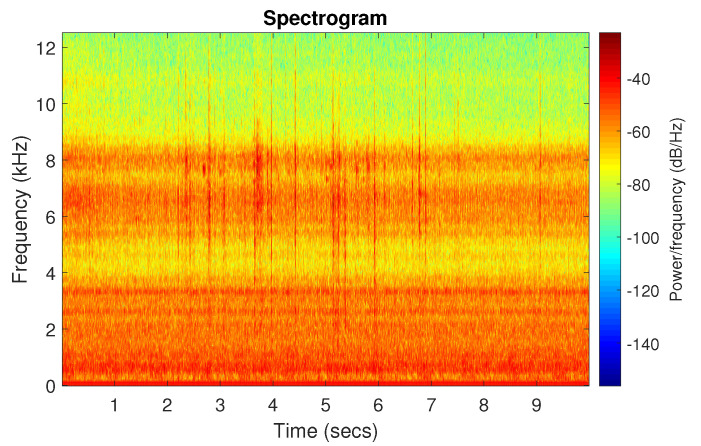
Spectrogram of the real signal r.

**Figure 41:**
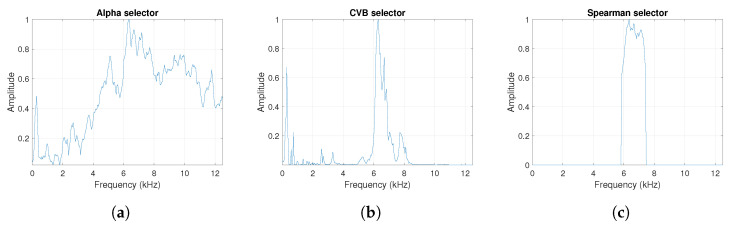
The considered IFB selectors for the real signal r. (**a**) Alpha selector. (**b**) CVB selector. (**c**) Spearman selector.

**Figure 42:**
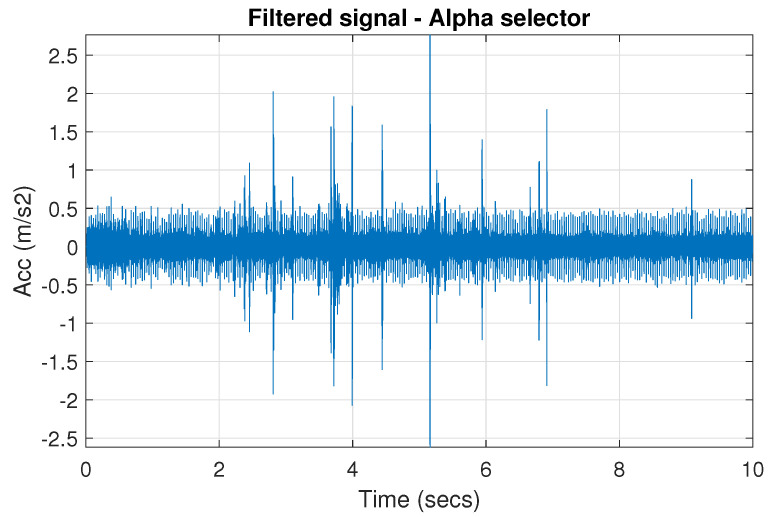
Filtered signal r by Alpha selector.

**Figure 43:**
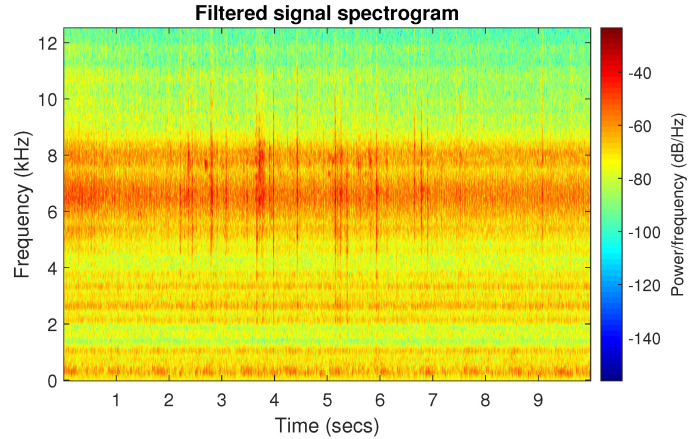
Spectrogram of filtered signal r by Alpha selector.

Figure 39 shows a vibration signal in the time domain, while its time-frequency counterpart is presented in Figure 40. Based on the spectrogram, the Alpha (Figure 41a), CVB (Figure 41b), and Spearman (Figure 41c) selectors are calculated. Although the Alpha selector correctly indicates the frequency band of cyclic impulses, it also takes high values in the frequency band corresponding to high-energy noise, i.e., at 5–11 kHz. The signal filtered with Alpha selector still have high non-cyclic amplitudes reaching the value 2.5; however, the amplitude of the remaining background noise is reduced by half and decreased to 0.5 (Figure 42). The high-energy noise, which remained in the signal is also visible in the spectrogram, which is shown in Figure 43.

**Figure 44:**
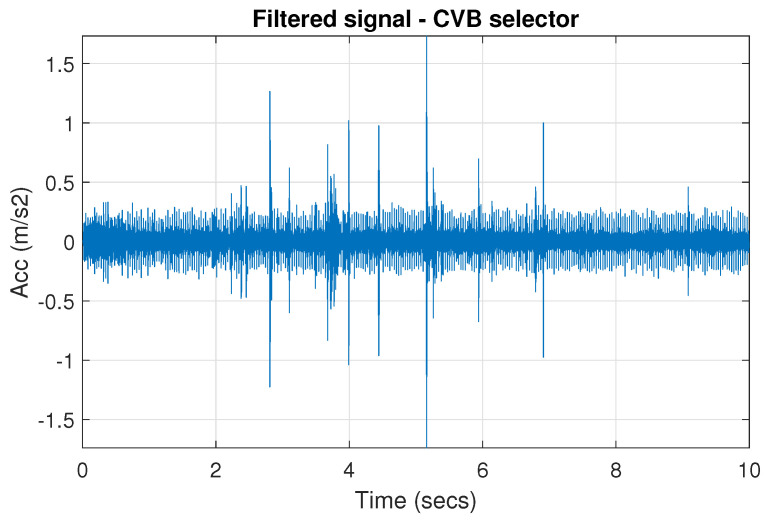
Filtered signal r by CVB selector.

**Figure 45:**
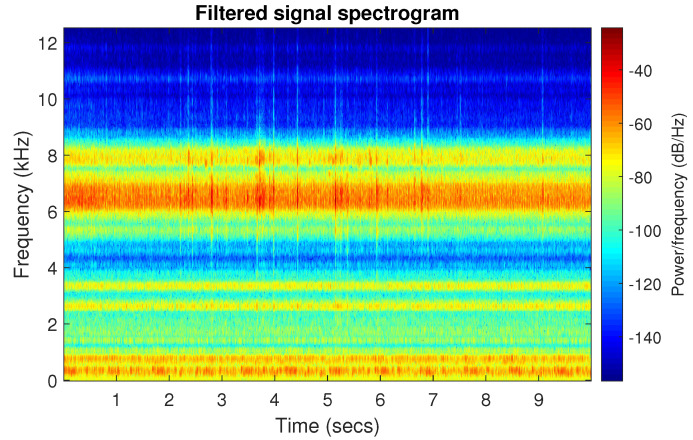
Spectrogram of filtered signal r by CVB selector.

**Figure 46:**
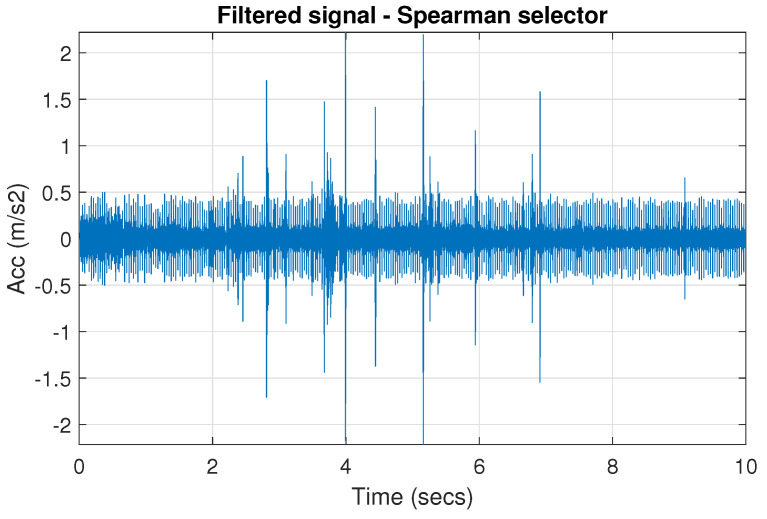
Filtered signal r by Spearman selector.

The CVB selector indicates the informative frequency band more distinctly, see Figure 44. It has high value in range of 6–7 kHz and some narrow bands of increased value at low frequencies. Although the fact that this selector exceeds 0.6 at 0.5 kHz, it has a very narrow frequency range there and it has little effect on the filtration result. Additionally, it indicates the bands with noise around 8 kHz. The amplitudes of the non-cyclic impulses of the signal after filtration with the CVB selector decreased to 1.6, the amplitudes of the remaining background noise is about 3 times smaller than for the real signal and reaches value 0.27, see (Figure 44). It results from the shape of the CVB selector, which indicates a narrower frequency band than the Alpha selector. The spectrogram of signal filtered by CVB selector is presented in Figure 45. The Spearman selector indicates the informative frequency band most unambiguously. As a result, the cyclic impulses are the most visible after filtration, see Figure 46. It is obvious that non-cyclic impulses are also noticeable, however they occur in every considered filtered signal. Selected frequency band with cyclic excitation covers also part of non-cyclic disturbances, because both components are spectrally overlapped, so it is clear that selection of frequency band cannot be ideal solution.

**Figure 47:**
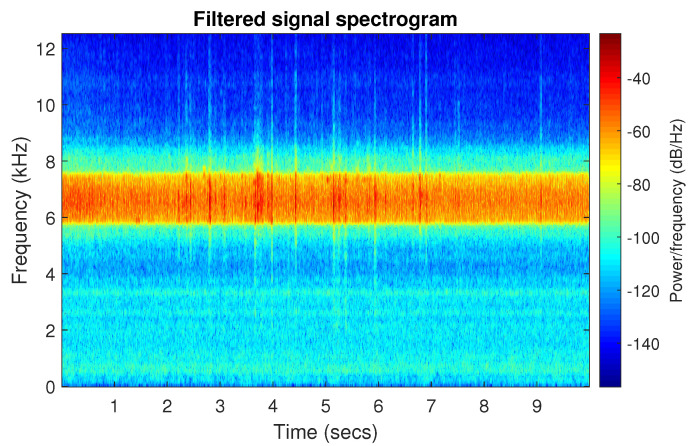
Spectrogram of filtered signal r by Spearman selector.

The good selectivity of the Spearman-based filter can also be seen on the spectrogram, see Figure 47. The high energy is focused only on the informative frequency band.

**Figure 48:**
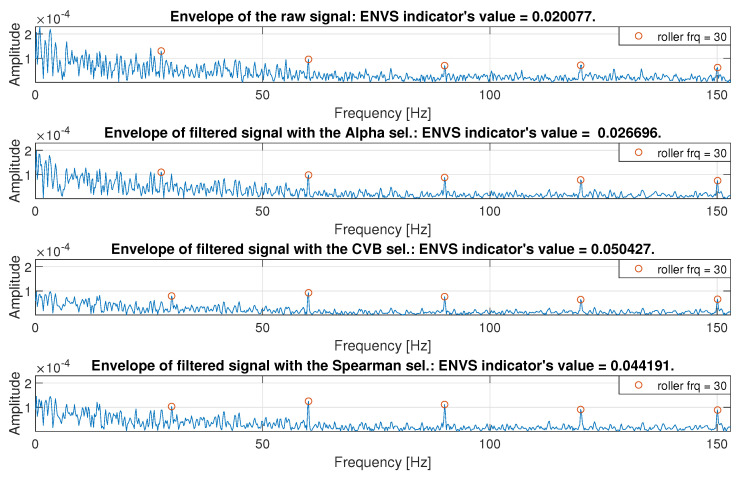
Envelope spectrum.

As a result that the analyzed signal is 10 s long, it is difficult to see the filtration effects and cyclic impulses associated to the fault, which are hidden in the long signal. Therefore, for comparison, the envelope spectrum (ES) is calculated for the raw signal and the signals after filtration, see Figure 48. As one can see, the energy of the ES until the first component related to fault (30 Hz) is significantly higher in the raw signal, as well as, in the signal filtered with the Alpha selector. Therefore, the component with fundamental fault frequency harmonic might be hardly detectable. The highest harmonics after Spearman filtration work to its advantage, but ES of the signal filtered with the CVB selector has lower background noise. This fact causes that the value of the ENVS indicator has slightly larger values in favor of CVB. Based on the graphical and numerical results, one may conclude that the CVB and Spearman selectors give comparable results in the considered case, that corresponds to the performed Monte Carlo simulations presented in the next section. However, considering the complexity of implementing both selectors, one may conclude that Spearman selector outperforms the CVB one.

## 5. Discussion

**Figure 49:**
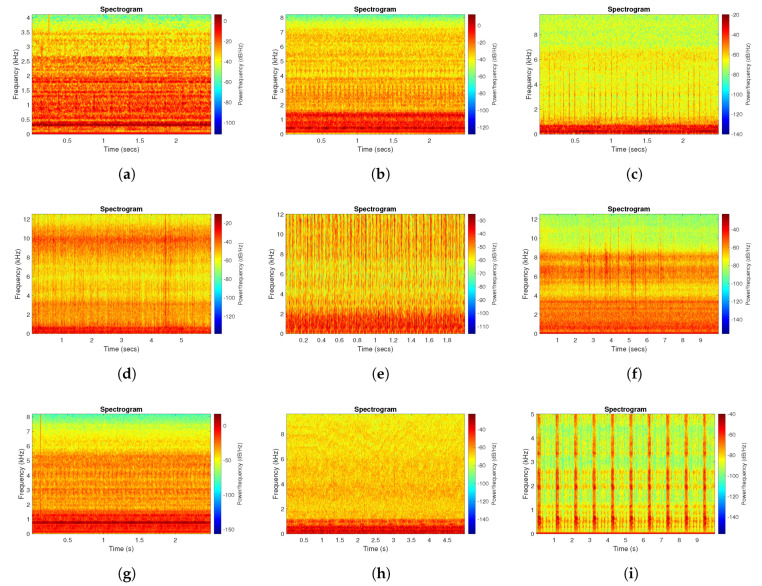
Spectrograms of various real signals. (**a**) Spectrogram of the real signal $r_{1}$ [56]. (**b**) Spectrogram of the real signal $r_{2}$ [57]. (**c**) Spectrogram of the real signal $r_{3}$ [5]. (**d**) Spectrogram of the real signal $r_{4}$ [44]. (**e**) Spectrogram of the real signal $r_{5}$ [28]. (**f**) Spectrogram of the real signal $r_{6}$. (**g**) Spectrogram of the real signal $r_{7}$. (**h**) Spectrogram of the real signal $r_{8}$. (**i**) Spectrogram of the real signal $r_{9}$ [58].

In the case of real machines, we have met various examples, that are presented in Figure 49. These different examples come from several types of machines: two-stage gearbox, bearings used in a pulley, crusher, oil compressor, or bearing from a paper cutting machine. In most of the cases, we have random broadband disturbances related to process or just acquired by accident. The presence of impulsive disturbance is clearly visible in the presented signals as they have much higher amplitudes than cyclic impulses. It may be a single impulse or series of impulses—dependent on the case. One may notice that the location of the informative band (cyclic impulses) may be very easy to detect or it is totally masked by the noise. As claimed earlier, widths of cyclic/non-cyclic disturbances are different, thus level of the spectral overlapping may be very different—the worst case is presented in Figure 49i, where wide-band disturbances associated with cutting totally dominate other components in the signal, see Table 2.

One may conclude that the problem we have defined exists in the real environment. To answer the question formulated in the Introduction (“How does it influence...?”) we proposed to use another numerical experiment. We do not consider the application of the proposed methodology for all real signals proposed in this section because a number of factors influencing analysis would make comparison very hard (i.e., ENVSI would be not appropriate to compare different machines). To properly validate our statement, one would need to consider the machine with several local damages (with different IFBs) and different levels of impulsive load. Therefore, the Monte Carlo simulations have been carried out to highlight the effectiveness of the algorithms and indicate the differences between the corresponding results depending on a carrier frequency of periodic impulses and amplitudes of non-periodic disturbances.

The plan of the experiment covers simulations and analysis of signals with amplitudes of non-periodic impulses taken from the set [5:3:35] and center frequencies of periodic impulses belonging to the set [2500:250:6000] Hz. The other parameters of the simulated signals are the same as we have considered in Section 3, namely the center frequency of the non-periodic impulses is 6000 Hz, and the amplitude of the periodic impulses is 3. More details about simulated signals have been presented in [34]. The ENVS indicator has been computed for filtered signals (by using three considered methods) to evaluate the efficiency of processing for each case. Let us note, that there is a varying degree of overlapping for cyclic and non-cyclic impulses in the range of 3000–4000 Hz. For the other considered cases, the frequency bands overlap totally. For each case, 1000 signals have been simulated.

**Figure 50:**
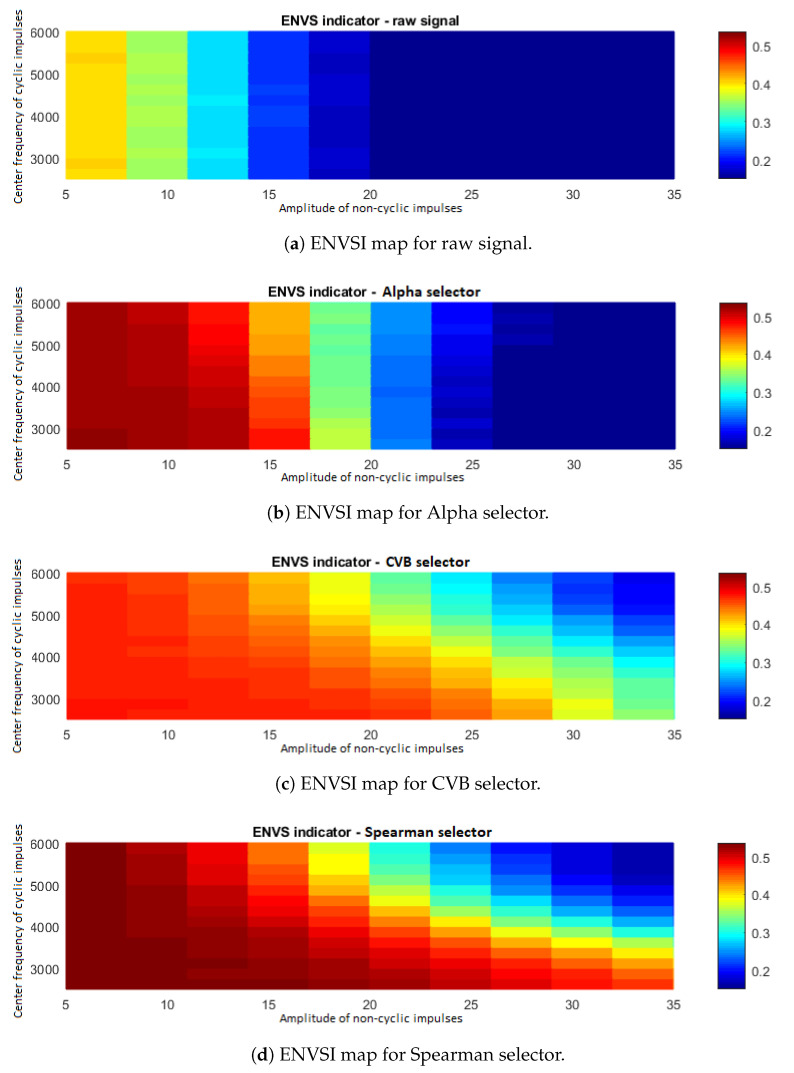
Maps with the mean of ENVS indicator values obtained by Monte Carlo simulation.

For each considered case (a combination of the parameters used for simulation) we calculate the mean value of ENVSI, see Figure 50. Figure 50a presents the ENVSI map for the raw signal. As expected, there are low values for all parameters. It is also not surprising that as for the decreasing amplitudes of non-cyclic impulses, the value of ENVSI increases.

Figure 50b illustrates the ENVSI map corresponding to the Alpha selector. As one can see, the ENVSI value indicates that the Alpha selector is independent of the center frequency of the periodic impulses and is sensitive only to the amplitude of the non-cyclic disturbances.

In Figure 50c, the ENVSI map for the signal filtered by the CVB selector has been presented. This map behaves similarly to the case of Spearman selector (see Figure 50d); however, the indicator values are lower than in the case of Spearman correlation-based method for most of the cases. In Figure 50d the map with the average ENVSI values for the filtered signal by the Spearman selector has been presented. Visually comparing this map with other considered cases (Figure 50b,c), it is noticeable that the high value of ENVSI appears most often. The Spearman selector works well regardless of the degree of the bands’ overlapping for non-cyclic amplitudes in the range of 5–18. For larger amplitudes of the non-cyclic disturbances, the ENVSI value depends on the level of overlapping—the greater the overlap of the bands corresponding to cyclic and non-cyclic impulses, the lower the ENVSI value.

**Figure 51:**
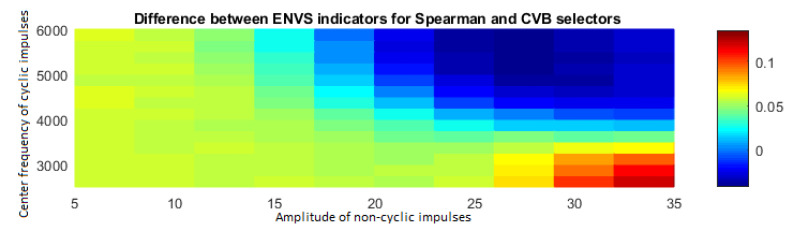
Difference between ENVS indicators for Spearman and CVB selectors.

Filtering with Spearman and CVB selectors is the most effective; therefore, for a more detailed comparison of these methods, a map containing the differences between the ENVSI values has been created–the CVB map has been subtracted from the Spearman map (see Figure 51).

**Figure 52:**
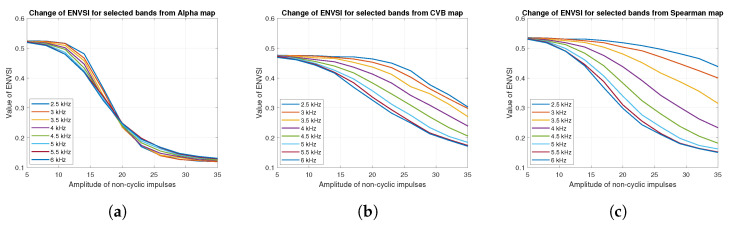
Change of ENVSI for selected frequency bands for the three considered methods: (**a**) Alpha selector, (**b**) CVB selector, (**c**) Spearman selector.

As one can see in Figure 51, the Spearman selector works better for most of cases. In particular, this method allows for a more precise analysis for cases where the amplitude of non-periodic impulses is higher than 25, and the center frequency of periodic impulses is in the range of 2.5–3.5 kHz, i.e., where frequency bands of cyclic and non-cyclic impulses do not completely overlap. However, when the amplitude of non-cyclic disturbances is in the range of 20–35 and the center frequency of cyclic impulses is in the range of 4–6 kHz, i.e., the frequency bands fully overlap, then the method based on CVB selector seems to be more accurate. Figure 52 shows the change of the ENVSI value depending on the amplitude of non-periodic impulses for different degrees of overlap between cyclic and non-cyclic impulses.

As mentioned, the overlap level does not affect the ENVSI value for the Alpha selector, see Figure 52a. For other cases, the smaller the degree of overlap between periodic and non-periodic impulses, the slower decrease of the ENVSI values with increasing amplitude (see Figure 52b,c).

## 6. Conclusions

The problem considered in this paper is the vibration-based local damage detection for signals with non-Gaussian-distributed noise. One of the main purposes of our research was to compare three recently proposed procedures of IFB selection at different levels of spectral overlapping corresponding to informative (signal of local fault, characterized by cyclic, impulsive behavior) and non-informative frequency bands (signal with non-cyclic impulses, not related to damage). The numerical experiment for four different cases (levels) of spectral overlapping has been proposed and a parameter based on the Envelope Spectrum-based Indicator (ENVSI) was used as a measure of effectiveness of selected diagnostic methods. The results for simulated signals clearly demonstrate that different levels of spectral overlapping for cyclic and non-cyclic impulses may significantly influence the diagnostic information. The numerical analysis show that for relatively high non-cyclic impulses, i.e., about five times larger than the cyclic ones, first, CVB and Spearman-based selectors are better than alpha-based selector, second, the greater the overlapping is, the smaller the ENVSI for the CVB and Spearman-based selector, that indicates the methods become less effective. In the worst case, these two selectors have comparable properties and the filtered signals takes the same ENVSI value. What is more, Alpha selector becomes much worse if the amplitudes of non-cyclic impulses are more than 5 times larger than the cyclic impulses regardless of the level of the spectral overlapping. It is the consequence of the high-energy impulses, which widely extend over a frequency band, which is what makes the Alpha selector become broadband. This effect has been observed during the analysis of simulated, as well as real vibration data.

For partial overlapping (or split frequency bands) the CVB and Spearman-based algorithms are more efficient than envelope for raw signal or Alpha-based filtering and in the particular cases the Spearman-based techniques gives the best results. For the highest overlapping and lowest amplitude of non-cyclic impulses, the Spearman-based method is only slightly better than the CVB-method. However, it is more intuitive and the algorithm is computationally simpler.

In the section devoted to the investigation of real data, we have analyzed the signal from the crushing machine. The best performing selectors for that case are the CVB and Spearman-based methods (due to the fact that we are dealing with 100% overlapping). This result corresponds to the simulation results presented in the paper, where the CVB and Spearman selector give better results than the Alpha-based algorithm. It should be mentioned, in the real case considered here, the ENVSI is slightly higher for CVB than for the Spearman method, but again, one can say that computational complexity is smaller and interpretation is easier for Spearman-based approach.

In the Discussion section, we have shown several examples of real signals for which the location of informative and non-informative frequency bands is different. Thus, the problem of spectral overlapping considered in this paper has an impact on the obtained results and it should be taken into account in the technical diagnostics. Finally, we have performed the Monte Carlo simulations, where different levels of spectral overlapping and different amplitudes of non-cyclic impulses were taken under consideration. The results of Monte Carlo simulations clearly indicate that the CVB and Spearman-based methods outperform the Alpha-based selector. For cases where the amplitude of non-periodic impulses is relatively small (<20) and the frequency bands of cyclic and non-cyclic impulses do not overlap completely, the Spearman selector outperforms the CVB algorithm. In very difficult cases—complete spectral overlapping and large amplitudes of non-cyclic impulses—CVB selector seems to be slightly better than the Spearman one.

In this article, we demonstrated the advantages and limitations of the considered methods in the practical applications. The analyzed in this paper problem is rarely discussed in the literature and thus the presented analysis seems to be unique in the technical diagnostics. In the further analysis, we plan to consider also the influence of the non-cyclic impulses frequency on the diagnostic procedures examined in this paper.

## Figures and Tables

**Table 1:** The numerical value of goodness of fault detection—Envelope Spectrum-based (ENVS) indicator values for different selectors and changing center frequency of the local fault.

Frequency of Cyclic Impulses [Hz]	ENVS Indicator Value		
	Alpha	CVB	Spearman
2500	0.1176	0.4231	0.5037
3500	0.2621	0.4930	0.4984
4000	0.1527	0.3812	0.3977
6000	0.3418	0.3471	0.3482

**Table 2:** The frequency ranges of cyclic and non-cyclic impulses for real signals.

Signal	Non-Periodic Impulses		Periodic Impulses
	Time [s]	Frequency [kHz]	Frequency [kHz]
$r_{1}—$ gear	0.25	0–0.45 and 2.7–4	0.8–1.2 also couple impulses around 3
$r_{2}—$ gear	0.1	7–8	2–5
$r_{3}—$ bearing	none	none	1–6
$r_{4}—$ bearing	whole signal, largest at 4.5 s	1–12	2–3.5
$r_{5}—$ bearing	whole signal	0–10	>8 (hardly visible)
$r_{6}—$ bearing	2–7	2–12	6–7.5
$r_{7}—$ gear	0.1	1.8–3.8 and 5.5–8	2.8–4.2
$r_{8}—$ bearing	0.5 and 1.4 and 3	c.a 3–7 and 4–6 and 3–8	none
$r_{9}—$ bearing	whole signal	whole band (cutting)	several sub-bands: 0–1 and 2–2.5
